# Genetic characterization of *Bacillus anthracis* in Guizhou Province, Southwest of China

**DOI:** 10.1186/s12866-015-0414-8

**Published:** 2015-03-31

**Authors:** Shijun Li, Qing Ma, Hong Chen, Dingming Wang, Ying Liu, Xiaoyu Wei, Lv You, Guanghai Yao, Kecheng Tian, Guangpeng Tang

**Affiliations:** Institute of communicable disease control and prevention, Guizhou Provincial Centre for Disease Control and Prevention, 101 Bageyan Road, Guiyang, 550004 Guizhou People’s Republic of China; Guiyang Centre for Animal Disease Control and Prevention, Guiyang, China

**Keywords:** Anthrax, *Bacillus anthracis*, MLVA, Genetic characterization

## Abstract

**Background:**

*Bacillus (B.) anthracis* is the pathogen that causes fatal anthrax. Guizhou Province is an old foci of anthrax in the southwest of China. Human anthrax has also been frequently reported in Guizhou in recent year. However, there is limited information on the genetic background of local *B. anthracis* isolates in Guizhou Province. Strain-specific detection of this bacterium using molecular approaches has enhanced our knowledge of microbial genetics. In the present study, we employed Multiple Locus Variable Number Tandem Repeats (VNTR) Analysis (MLVA) assay to analyze the genetic characteristics of *B. anthracis* strains isolated in Guizhou Province and their relationships to worldwide distributed isolates.

**Results:**

A total of 32 isolates of *B. anthracis* from soil, human, cattle, dog and water of different anthrax epidemics in Guizhou Province from 2006 to 2011 were confirmed with phage lysis test, penicillin inhibition test and PCR. MLVA-8 discriminated them into 28 unique MLVA types (MT G1 - G28), which were novel MTs compared with the previous reports. Cluster tree based on 32 isolates from Guizhou Province and 76 worldwide distributed isolates (30 MTs) showed they were divided into three clusters, designated A, B and C. All the 32 isolates were distributed in cluster A, which were further grouped into A1, A2, A3 and A4 sub-clusters.

32 isolates from Guizhou Province were closely grouped in each of the sub-clusters, respectively. Minimum Spanning Tree (MST) based on the MLVA data showed that the 28 MLVA profiles of isolates from Guizhou Province and 30 MLVA profiles of worldwide distributed isolates formed three clonal complexes (CCs) and ten singletons.

**Conclusions:**

28 novel MTs of *B. anthracis* from Guizhou were revealed and their relationships to worldwide isolates were showed. The results will provide important information for prevention of anthrax and also enhances our understanding of genetic characteristics of *B. anthracis* in China.

## Background

Anthrax is an often fatal bacterial infection that occurs when *Bacillus anthracis* endospores enter the body through abrasions in the skin or by inhalation or ingestion [1,2]. It is a zoonosis to which most mammals, especially grazing herbivores, are considered susceptible [2]. While anthrax currently affects mostly livestock and wildlife around the world, it can and does kill humans [3]. Humans almost invariably contract anthrax from handling infected animals or carcasses of animals that have died of the disease, or meat, skins, hair, bones, etc. from such animals [4]. Anthrax infection in humans occurs by three major routes, the skin, the respiratory tract or the gastro-intestinal tract, generating three different primary forms of the disease, the cutaneous, the inhalational and the gastro-intestinal forms [2]. Cutaneous anthrax, the most common form, occurs as a result of contamination of skin with the bacterial spores, due to its mechanical abrasion or damage caused by insect bites [5]. Besides, the great current interest in anthrax is due to its potential as a bioterrorism and biowarfare agent [6,7].

Ancient Chinese medical books suggest that an anthrax-like disease has been present in China for more than 5,000 years and the epidemiology and symptoms of anthrax had been described [8]. An anthrax surveillance and control project reported 72 outbreaks and 8,988 human cases in ten provinces (Sichuan, Tibet, Inner Mongolia, Xinjiang, Qinghai, Gansu, Guangxi, Guizhou, Yunnan and Hunan) in China in 1990–1994, which indicates a long history of anthrax epidemics in China [8]. More recent reports showed the sporadic epidemics or outbreaks of anthrax in many provinces of China, such as, Mongolia, Liaoning and Jiangsu [9-11].

Guizhou Province, with nearly 50 million people, is an old foci of anthrax in the southwest of China. For example, a total of 17,975 cases of anthrax in Guizhou Province from 1957 to 1999 had been reported, and the epidemics covered all of the nine prefectures, including Guiyang, Qianxinan, Qiandongnan, Qiannan, Anshun, Bijie, Liupanshui, Tongren and Zunyi, in Guizhou Province [12]. Human anthrax has also been frequently reported in Guizhou in recent year. For instance, there were 32 outbreaks of anthrax occurred in Guzhou Province [13]. An more recent outbreak of cutaneous anthrax including seven cases has been reported in a village of Wangmo County in Guizhou Province in 2010 [14]. However, there is limited information on the genetic background of local *B. anthracis* isolates in Guizhou Province. In this study, we applied MLVA-8 to analyze the genetic characteristics of *B. anthracis* strains isolated from Guizhou Province.

## Methods

### Bacterial isolates and confirmation

A total of 32 of isolates were used for analysis in this study (Table 1). All of these isolates were come from the local epidemics of cutaneous anthrax in Guizhou Province from 2006–2011, which included isolates from human and epidemiologic related animal (dog and cattle), soil and water in each epidemic. The localities covered Prefecture Qianxinan, Qiannan, Anshun, Tongren, and Bijie. All the isolates used in this study were confirmed with PCR kit (Takara, Janpan) and conventional methods including phage lysis test, penicillin inhibition test for anthrax diagnosis.

**Table 1:** **Backgroud information and pathogenic plasmid content of** ***B. anthracis*** **isolates used in this study**

**Strains**	**Original no.**	**Source**	**Prefecture**	**County**	**Year**	**pXO1**	**pXO2**
GZBA01	2006001	Dog	Qianxinan	Ceheng	2006	+	+
GZBA02	2006002	Dog	Qianxinan	Ceheng	2006	+	+
GZBA03	2006003	Soil	Qianxinan	Zhenfeng	2006	+	+
GZBA04	2006004	Human	Qianxinan	Zhenfeng	2006	+	+
GZBA05	2006005	Soil	Qianxinan	Zhenfeng	2006	+	+
GZBA06	2006006	Soil	Qianxinan	Zhenfeng	2006	+	+
GZBA07	2007001	Soil	Qianxinan	Ceheng	2007	+	+
GZBA08	2007003	Soil	Anshun	Zhenning	2007	+	+
GZBA09	2007004	Soil	Qiannan	Luodian	2007	+	+
GZBA10	2007005	Soil	Anshun	Zhenning	2007	+	+
GZBA11	2007007	Water	Qianxinan	Ceheng	2007	+	+
GZBA12	2007008	Soil	Qianxinan	Ceheng	2007	+	+
GZBA13	2007013	Soil	Qianxinan	Ceheng	2007	+	+
GZBA14	2007018	Soil	Qianxinan	Zhenfeng	2007	+	+
GZBA15	2007019	Soil	Qianxinan	Ceheng	2007	+	+
GZBA16	2007022	Soil	Qianxinan	Ceheng	2007	+	+
GZBA17	2007023	Soil	Qiannan	Luodian	2007	+	+
GZBA18	2007024	Soil	Qianxinan	Ceheng	2007	+	+
GZBA19	2007029	Soil	Qiannan	Luodian	2007	+	+
GZBA20	2008001	Water	Qianxinan	Ceheng	2008	+	+
GZBA21	2008002	Soil	Qiannan	Duyun	2008	+	+
GZBA22	2009001	Soil	Qiannan	Guiding	2009	+	+
GZBA23	2009002	Soil	Bijie	Dafang	2009	+	+
GZBA24	2009003	Soil	Bijie	Zhijin	2009	+	+
GZBA25	2009024	Soil	Qianxinan	Zhenfeng	2009	+	+
GZBA26	2010001	Soil	Qianxinan	Anlong	2010	+	+
GZBA27	2010002	Soil	Qianxinan	Wangmo	2010	+	+
GZBA28	2010007	Soil	Qianxinan	Wangmo	2010	+	+
GZBA29	2010010	Cattle	Qianxinan	Wangmo	2010	+	+
GZBA30	2010011	Soil	Qianxinan	Anlong	2010	+	+
GZBA31	2011002	Soil	Tongren	Sinan	2011	+	+
GZBA32	2011003	Soil	Qianxinan	Zhenfeng	2011	+	+

### DNA templates preparation

DNA templates for PCR were prepared directly from bacterial colonies by the boiling method [3]. Briefly, *B. anthracis* cells were streaked onto blood agar plates and then incubated at 37°C overnight. A single colony from each plate was transferred into a microcentrifuge tube containing 100 μl of DNA extraction liquid (Liferiver, China). The colony was resuspended by vortexing or repetitive pipetting. The cellular suspension was heated to 95°C for 20 min and then cooled to room temperature. Cellular debris was removed by centrifugation at 15,000 g for 5 min. The supernatant, containing DNA, was used as the template for PCR amplification.

### MLVA experiments

For the MLVA test, we utilized primers that flank the eight VNTR regions (vrrA, vrrB1, vrrB2, vrrC1, vrrC2, CG3, pXO1, pXO2) as described by previous study [3,15]. Amplifications were performed in 50 μl total volumes of PCR reaction system contained approximately 25 μl of PreMix Taq (TaKaRa, Otsu, Japan), 2 μl of forward and reverse primers with concentrations of 10 pmol/μl, 2 μl of DNA, 19 μl of deionized water, respectively. Amplification was performed on an Biometra TProfession thermocycler (Biometra, Goettingen, Germany) using amplification parameters included an initial denaturation at 94°C for 5 minutes, followed by 34 cycles of 94°C for 20 seconds, 60°C for 20 seconds, 65°C for 20 seconds, then 65°C for 5 minutes. PCR products were detected by electrophoresis of 1 μl of each reaction on a 1.2% agarose gel for 30 min at 100 V, and were sequenced by ABI PRISM 377 DNA sequencer. The full length of each locus were obtained by assembling of forward and reverse sequences using ContigExpress (Invitrogen Life Science, Carlsbad, CA), on which the forward sequence and the reverse complement sequence of reverse primer were marked to determine the actual length of amplification. The actual length were calibrated to the nearest sizes for the corresponding VNTR in the report of Keim *et al.* [3].

### Data analysis

Each unique profile based on allelic combination was designated a unique MLVA type (MT). The Bionumerics software package, version 4.0 (Applied Maths, Belgium) was used for UPGMA clustering analysis based on categorical coefficient, and minimum spanning tree algorithm was used to construct a minimum spanning tree (MST) to determine phylogenetic pattern. The MLVA data of isolates of other provinces in China and worldwide isolates were come from *B. anthracis* data base (http://mlva.u-psud.fr/MLVAnet/spip.php?article123).

### Ethic statements and biosafety containment

All experiments involving *B. anthracis* including isolates from human, animal and environments were performed according to the General Requirements for Bio-safety (GB 19489–2008) and approved by the Biosafety Committee of Guizhou Provincial Centre for Disease Control and Prevention, and were also approved by the Ethics Review Committee of Guizhou Provincial Centre for Disease Control and Prevention.

## Results

### Bacteria distribution and confirmation

All the 32 isolates of *B. anthracis* were confirmed with phage lysis test, penicillin inhibition test and PCR (Table 1). Among these isolates, 26 isolates were isolated from soil, with one from human, one from cattle, two from dog and two from water. According to the year of isolation, six isolates were isolated in 2006, with 13 from 2007, six from 2008, four from 2009, five from 2010 and two from 2011. Based on the origin of region, 21 isolates were from Prefecture Qianxinan with two from Anshun, five from Qiannan, two from Bijie and one from Tongren.

### MLVA based genotypes

**Figure 1:**
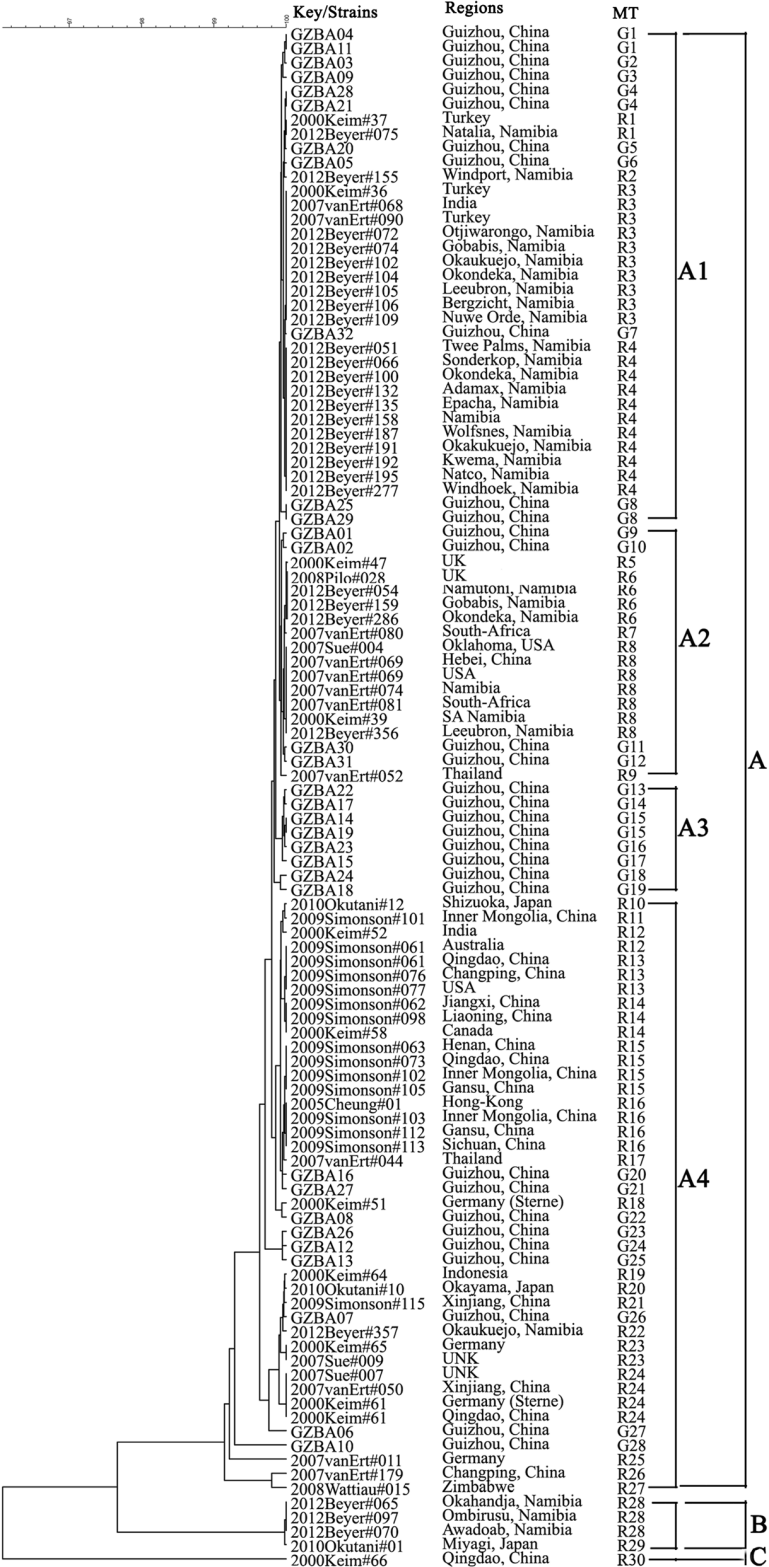
**Genetic relationships based on cluster tree of the 28 MLVA profiles of** ***B. anthracis*** **isolates from Guizhou province and isolates of worldwide distribution.** The MLVA data of of isolates from other province of China and isolates of worldwide distribution were come from the *B. anthracis* data base (http://mlva.u-psud.fr/MLVAnet/spip.php?article123). The dendrogram based on the MLVA profiles of isolates from Guizhou Province and isolates of worldwide distribution were constructed using UPGMA.

MLVA based on eight VNTR loci were performed to characterize the local isolates of *B. anthracis* in Guizhou Province. The copy numbers of each VNTR locus are listed in Table 2. The 32 *B. anthracis* isolates, based on the unique MLVA profiles, were discriminated into 28 unique MLVA types (MTs), which were nominated as MT G1 - G28 (Figure 1). Among the 28 MTs, MT G1, G5, G13 and G19 included two isolates, respectively, while each of the remaining 24 MTs only contained one isolates. Further, all the MLVA profiles based on eight loci were different from the 30 represent profiles (MT R1 - R30) of *B. anthracis* from other provinces in China and other countries.

**Table 2:** **Observed fragment sizes and copy numbers at eight MLVA loci in the** ***B. anthracis*** **strains isolated in Guizhou Province**

**Strains**	**Original no.**	**Year**	**MLVA profiles**															
			**vrrA**		**vrrB1**		**vrrB2**		**vrrC1**		**vrrC2**		**CG3**		**pXO1-aat**		**pXO2-at**	
			**S**	**N**	**S**	**N**	**S**	**N**	**S**	**N**	**S**	**N**	**S**	**N**	**S**	**N**	**S**	**N**
GZBA01	2006001	2006	325	11	229	16	162	7	581	53	532	17	168	4	135	11	149	14
GZBA02	2006002	2006	325	11	229	16	162	7	581	53	514	16	168	4	132	10	149	14
GZBA03	2006003	2006	325	11	229	16	162	7	581	53	532	17	168	4	135	11	143	11
GZBA04	2006004	2006	325	11	229	16	162	7	581	53	532	17	168	4	135	11	145	12
GZBA05	2006005	2006	325	11	229	16	171	8	581	53	532	17	168	4	135	11	143	11
GZBA06	2006006	2006	325	11	229	16	189	10	581	53	532	17	168	4	138	12	143	11
GZBA07	2007001	2007	313	10	229	16	162	7	581	53	532	17	163	3	132	10	139	9
GZBA08	2007003	2007	313	10	229	16	189	10	581	53	532	17	163	3	132	10	145	12
GZBA09	2007004	2007	325	11	229	16	162	7	581	53	532	17	168	4	138	12	143	11
GZBA10	2007005	2007	325	10	229	16	162	7	581	53	532	17	163	3	147	15	143	11
GZBA11	2007007	2007	325	11	229	16	162	7	581	53	532	17	168	4	135	11	145	12
GZBA12	2007008	2007	349	13	229	16	162	7	581	53	532	17	158	2	135	11	143	11
GZBA13	2007013	2007	325	11	229	16	162	7	581	53	532	17	163	3	133	10	143	11
GZBA14	2007018	2007	313	10	229	16	162	7	581	53	532	17	158	2	138	12	143	11
GZBA15	2007019	2007	325	11	229	16	162	7	581	53	532	17	158	2	138	12	145	12
GZBA16	2007022	2007	313	10	238	17	162	7	581	53	532	17	163	3	132	10	143	11
GZBA17	2007023	2007	313	10	229	16	162	7	581	53	532	17	158	2	135	11	143	11
GZBA18	2007024	2007	313	10	229	16	162	7	581	53	532	17	158	2	138	12	149	14
GZBA19	2007029	2007	313	10	229	16	162	7	581	53	532	17	158	2	138	12	143	11
GZBA20	2008001	2008	313	10	229	16	162	7	581	53	532	17	163	3	132	10	143	11
GZBA21	2008002	2008	313	10	229	16	171	8	581	53	532	17	163	3	132	10	143	11
GZBA22	2009001	2009	313	10	238	17	162	7	581	53	532	17	158	2	135	11	143	11
GZBA23	2009002	2009	313	10	238	17	162	7	581	53	532	17	158		138	12	143	11
GZBA24	2009003	2009	313	10	229	16	171	8	581	53	514	16	158	2	138	12	143	11
GZBA25	2009024	2009	325	11	229	16	162	7	581	53	532	17	158	2	132	10	145	12
GZBA26	2010001	2010	349	13	229	16	162	7	581	53	532	17	158	2	132	10	145	12
GZBA27	2010002	2010	325	11	229	16	162	7	581	53	543	17	158	2	132	10	143	11
GZBA28	2010007	2010	313	10	229	16	171	8	581	53	532	17	163	3	132	10	143	11
GZBA29	2010010	2010	325	11	229	16	162	7	581	53	532	17	158	2	132	10	145	12
GZBA30	2010011	2010	313	10	229	16	162	7	581	53	532	17	158	2	132	10	147	13
GZBA31	2011002	2011	313	10	229	16	162	7	581	53	532	17	158	2	135	11	147	13
GZBA32	2011003	2011	313	10	229	16	162	7	581	53	532	17	163	3	132	10	145	12

**S:** fragment sizes (bp); N: copy numbers.

### Genetic relationship based on clustering analysis

MLVA data of 32 isolates from Guizhou province and 76 worldwide distributed isolates belonging to 30 MTs (MT R1 to 30), which includes 19 isolates (9 MTs) from 11 other provinces of China (Hebei, Inner Mongolia, Qingdao, Changping, Jiangxi, Liaoning, Henan, Gansu, Sichuan, Xinjiang, Hong-Kong) and 57 isolates (21 MTs) of 14 other countries (Australia, Canada, Germany, India, Indonesia, Japan, Namibia, South-Africa, Thailand, Turkey, UK, UNK, USA, and Zimbabwe) were used for comparison. The cluster tree based on isolates from Guizhou Province and worldwide distributed isolates showed they were divided into three clusters (Figure 1), designated A, B and C. All the 32 isolates were distributed in cluster A, which were further divided into A1, A2, A3 and A4 sub-clusters. MT G1 - G8 isolates of Guizhou Province and MT R1-R4 isolates from India, Namibia and Turkey formed sub-cluster A1, in which isolates from Guizhou Province were relatively closely clustered. Sub-cluster A2 contains 18 isolates from Guizhou and Hubei Province of China, UK, South-Africa and Namibia. Isolates of MT G13-G19 from Guizhou Province formed the sub-cluster A3, while sub-cluster A4 includes 42 isolates (26 MTs) from China (including Guizhou, Inner Mongolia, Qingdao, Changping, Jiangxi, Liaoning, Henan, Gansu, Hongkong, Sichuan, Xinjiang Province) and other Countries, which included Asian countries such as Japan, Thailand and Indonesia and US, Germany, Zimbabwe, UNK and Namibia. It also showed that isolates from most of the Asian countries were grouped in sub-cluster A4, in which isolates from Guizhou were closely clustered. Four isolates from Zimbabwe, Namibia and Japan formed Cluster B, while isolates from Qingdao Province of China was separately formed the cluster C.

### Genetic relationship based on minimum spanning tree

**Figure 2:**
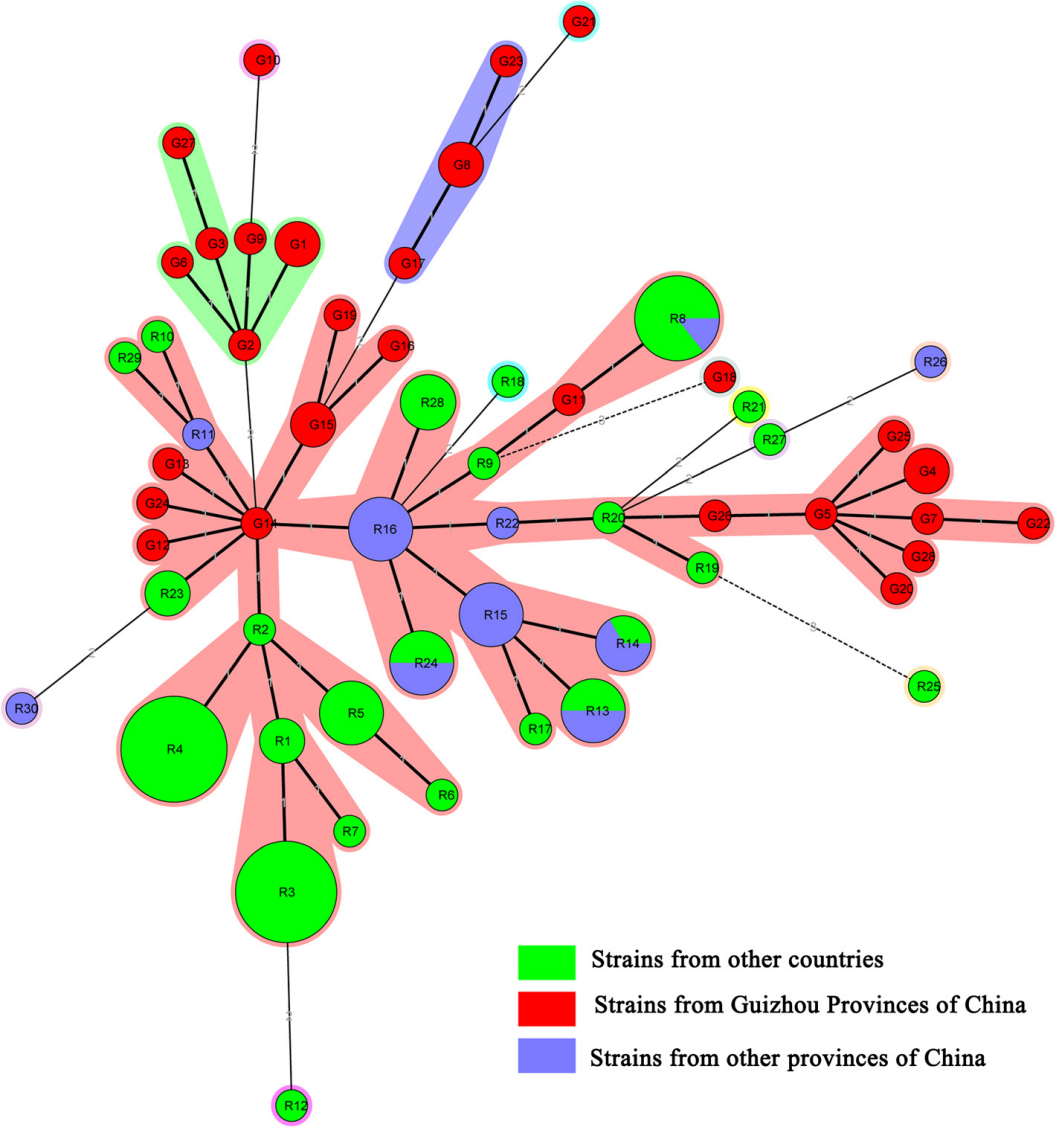
**Genetic relationships based on minimum spanning tree of the 28 MLVA profiles of** ***B. anthracis*** **isolates from Guizhou province and isolates of worldwide distribution.** Each circle corresponds to a MLVA profiles. The shadow zones in different colour correspond to different clonal complexes. The size of the circle is proportional to the number of the isolates, and the colour within the cycles represents the locality origin of Guizhou Province (red), other provinces of China (blue) or other countries (green).

Minimum Spanning Tree (MST) based on the MLVA data showed that the 28 MLVA profiles of isolates from Guizhou Province and 30 MLVA profiles of worldwide distributed isolates formed three clonal complexes (CCs) (G2, G8 and R16) and ten singletons (G10, G18, G21, R12, R18, R21, R25, R26, R27 and R30). All of isolates in CC G2 and CC G8 were come from Guizhou Province, while CC G14 contained isolates from both China (including Guizhou Province and other provinces of China) and other countries, in which isolates of MT G12, G14, G15, G18, G19 and G24 from Guizhou Province of China were relatively closely related, and the relationship of isolates of MT G4, G5, G7, G16, G20, G22, G25, G26 and G28 were relatively close in CC R16, which contained isolates of worldwide distribution (Figure 2).

## Discussion

*B. anthracis* is a member of the *Bacillus cereus* group, containing *Bacillus cereus*, *Bacillus thuringiensis*, *Bacillus mycoides* and *B. anthracis* [16]. *B. anthracis* spores can remain stable for decades. This same longevity may greatly influence the ecology and evolution of this pathogen. The resting stage probably greatly reduces the rate of evolutionary change, and this may contribute to the extremely homogeneous nature of *B. anthracis* [3,17].

Numerous studies have demonstrated the lack of molecular polymorphism within *B. anthracis*, which complicates efforts to subtype it [3,17-19]. The widely used molecular methods, such as Pulsed-field gel electrophoresis (PFGE) and multiple-locus sequence typing (MLST), often do not distinguish among closely related species, and many strains within a species show identical patterns (e.g., *Bacillus* cereus and *B. anthracis*) [3,20]. MLVA based on eight marker loci (MLVA-8) has been proved an effective molecular tool for discriminating among different *B. anthracis* isolates [3,15,21-23]. Therefore, we chose MLVA-8 to analyze the genetic characteristics of *B. anthracis* strains isolated in Guizhou Province and their relationship to strains of worldwide distribution. Anthrax is an important zoonosis in Guizhou Province in the history and it was first reported in Guizhou Province in 1957 [12,13]. Untill 1980s, the incidence in Guizhou Province exceeded the average in China and became the province with high incidence in 1990s, with incidence of 47.6% in 1991 [12]. Although the incidence reduced in recent year, sustained anthrax has been reported in recent years [13,14] and it was still more than the average level in China. At the same time, anthrax in animal is also a sever disease and caused sever economic lost in Guizhou Province [24]. The nationwide control system including disease outbreak surveillance has been carried out in Guizhou in the past few years and we recovered *B. anthracis* isolates from different epidemics. However, information on the genetic relationships of circulating *B. anthracis* isolates from Guizhou Province is lacking. Therefore, a total of 32 strains isolated from the local epidemics of cutaneous anthrax in Guizhou Province from 2006 to 2011 were used for analysis in this study. The results indicated that 32 *B. anthracis* isolates were discriminated into 28 MTs, among which only four MTs (MT G1, G5, G13 and G19) were represent by more than one isolates, while the remaining MTs contained only one isolates. This suggested the genetic diversity of *B. anthracis* in Guizhou Province.

In order to analyse the relation of strains from Guizhou Province to strains of worldwide distribution, we compared each MLVA-8 profiles of the 32 isolates in the *B. anthracis* data base (http://mlva.u-psud.fr/MLVAnet/spip.php?article123) to query identical MLVA profile in reported strains, and on identical profile was found in the online data base, which indicated that the 28 MLVA profiles of isolates from Guizhou Province were novel. Moreover, we downloaded MLVA-8 data of isolates from other provinces of China and other countries from the *B. anthracis* data base for cluster analysis, a total of 76 worldwide distributed isolates belonging to 30 MTs inluding 19 isolates (9 MTs) from 11 other provinces of China and 57 isolates (21 MTs) of other countries were used for comparison. Cluster tree suggested that all the 32 isolates from Guizhou Province were distributed in cluster A (Figure 1) that can be further grouped into A1, A2, A3 and A4 sub-clusters, and most of isolates from Guizhou Province were relatively closely clustered in each sub-cluster, which suggested the relatively close relations of isolates from Guizhou Province. Besides, isolates of Chinese (including Guizhou and other provinces) and other Asian countries were grouped in sub-cluster A4, which suggested the relatively close relationship of isolates from Asian countries. Besides, MST tree clearly showed the relations of isolates of Guizhou Province and worldwide, in which two CCs (CC G2 and G8) contained isolates only from Guizhou Provinces, while CC R16 contained isolates from different countries, in which isolates from Guizhou Province were much more related. Our results will not only provide important information for prevention of anthrax and also enhances our understanding of genetic characteristics of *B. anthracis* in China.

## Conclusions

A total of 32 isolates of *B. anthracis* from different anthrax epidemics in Guizhou Province during 2006 to 2011 were analysed using MLVA-8. 28 novel MTs of *B. anthracis* from Guizhou were revealed and the relationship to worldwide isolates was showed. The results will provide important information for prevention of anthrax and also enhances our understanding of genetic characteristics of *B. anthracis* in China.
