# Correction to: Genetic characterization of *Bacillus anthracis* in Guizhou Province, Southwest of China

**DOI:** 10.1186/s12866-018-1172-1

**Published:** 2018-04-12

**Authors:** Shijun Li, Qing Ma, Hong Chen, Dingming Wang, Ying Liu, Xiaoyu Wei, Lv You, Guanghai Yao, Kecheng Tian, Guangpeng Tang

**Affiliations:** 1 Institute of communicable disease control and prevention, Guizhou Provincial Centre for Disease Control and Prevention, Guiyang, People’s Republic of China; 2 Guiyang Centre for Animal Disease Control and Prevention, Guiyang, China

## Erratum

Upon publication of the original article [1] it was highlighted by the authors that a grant awarded to support the research work of the study was missed in the acknowledgements. It should also be acknowledged that the grant titled *“Genotyping and Molecular Epidemiological Characteristic of Bacillus anthracis in Guizhou Province”* awarded by the Program of Natural Science Foundation of Guizhou Province (Grant No. Qian Ke He J Word [2015] 2084) also contributed to the resources for this research. This has since been formally noted in this correction article.
